# Modulation of miR-155-5p signalling via 5-ASA for the prevention of high microsatellite instability: an in vitro study using human epithelial cell lines

**DOI:** 10.1007/s13105-024-01033-y

**Published:** 2024-07-10

**Authors:** Monika Adamowicz, Joanna Abramczyk, Ewa Kilanczyk, Piotr Milkiewicz, Alicja Łaba, Malgorzata Milkiewicz, Agnieszka Kempinska-Podhorodecka

**Affiliations:** 1 Department of Medical Biology, Pomeranian Medical University in Szczecin, Szczecin, 70-111 Poland; 2 Liver and Internal Medicine Unit, Medical University of Warsaw, Warszawa, Poland; 3 Translational Medicine Group, Pomeranian Medical University in Szczecin, Szczecin, Poland

**Keywords:** miR-155, Mismatch repair genes, Aminosalicylic acids, Primary sclerosing cholangitis

## Abstract

5-aminosalicylic acid (5-ASA) is a first-line treatment for maintaining colitis remission. It is a highly effective, safe, and well-tolerated drug with anti-inflammatory and chemo-preventive properties. While patients with primary sclerosing cholangitis (PSC) with concomitant ulcerative colitis are treated with 5-ASA, the molecular mechanisms underlying the drug’s chemo-preventive effects are not entirely understood. We previously reported that bile acids and lipopolysaccharide-induced miR-155 expression was associated with downregulating mismatch repair (MMR) proteins in CACO-2 cell lines. Therefore, in this investigation, a set of in vitro functional studies was performed to show the possible mechanisms behind the epigenetic relationship between miR-155 and 5-ASA’s prevention of high microsatellite instability (MSI-H). In transient transfection with miR-155Mimic, which behaves like endogenous miRNA, we confirmed the relationships between miR-155 and its target MMR in three human intestinal epithelial cell lines: CACO-2, NCM460D and HT-29. We have shown, for the first time, that 5-ASA modulates MLH1, MSH2, MSH6 in miR-155 transfected cells. These findings underline that chemoprotective 5-ASA therapy can effectively attenuate the expression of miR-155 and potentially prevent a development of MSI-H in a subset of colorectal cancers associated with PSC.

**Supplementary Information:**

The online version contains supplementary material available at 10.1007/s13105-024-01033-y.

## Key Points

- miR-155 may be a key regulator of tumorigenesis in the ascending colon of patients with PSC.
- 5-ASA therapy can effectively attenuate the expression of miR-155 involved in the pathogenesis of high microsatellite instability.

**Supplementary Information:**

The online version contains supplementary material available at 10.1007/s13105-024-01033-y.

## Introduction

Colorectal carcinoma (CRC) is not a uniform disease, although it can be distinguished by a range of genomic and epigenomic modifications [1]. Recently, the mechanism underlying CRC tumorigenesis has been linked to miRNAs [2]. These are a class of small (~ 20 nucleotides), endogenous, non-coding RNAs that modulate gene expression by binding to the 3’-UTR of the target mRNA, leading to either its degradation or repression of protein translation. miRNAs may possess either tumour-suppressive or oncogenic activity, depending on their target genes [3]. The important role of miRNAs in the immune response has been highlighted by studies in which the deregulation of miRNAs was shown to accompany diseases involving excessive or uncontrolled inflammation [4]. However, research into the role of miRNAs in the pathogenesis of susceptibility to colon carcinogenesis in patients suffering from cholestatic liver disease such as primary sclerosing cholangitis (PSC) is insufficient. In the course of PSC disease, bile duct enlargement, fibrosis and inflammatory infiltration of the extrahepatic and intrahepatic bile ducts have been observed [5]. The disorder that most commonly accompanies PSC is ulcerative colitis (UC). The presence of PSC with concomitant UC (PSC/UC) substantially raises the risk of colon cancer [6]. This risk is thought to be 4–10 times greater than the risk of developing CRC in patients with UC without PSC, and it develops at a much younger age than in patients with UC alone. Furthermore, in the majority of PSC/UC patients who developed CRC, the tumours are located on the right side of the colon, unlike in patients with UC alone, where the tumours occur more frequently on the left side of the colon [7]. Two main mechanisms by which colorectal cancer develop is chromosomal instability (CIN) that includes loss of APC, 18q and p53, or microsatellite instability (MSI) which occurs in approximately 15% of sporadic colorectal cancers [8, 9].

Previous studies have suggested that miR-155 functions as an oncogenic miRNA in human cancers [10]. High expression levels of miR-155 have been found to correlate with the poor prognosis of colorectal cancer [11], and, depending on its target genes, miR-155 can potentiate oncogenic activity in the colon [12, 13]. In our previous studies, we have suggested that miR-155 is a key regulator of tumorigenesis in patients with PSC [14, 15]. Moreover, miR-155 can regulate the expression of mismatch repair (MMR) genes to influence genomic stability in CRC [16]. MMR proteins are nuclear enzymes that form heterodimers that bind to areas of abnormal DNA and initiate its removal. Loss of MMR proteins leads to the accumulation of DNA replication errors, which is termed microsatellite instability (MSI) [17]. The term MSI-H refers to high microsatellite instability in which > 30% of the microsatellite marker panel is mutated (two or more of the five markers, i.e., MLH1, MSH2, MSH6, PMS1 or PMS2) [18, 19]. In our previous study, we found a correlation between the upregulation of miR-155 and the downregulation of MMR genes in different parts of the colon in PSC patients [14, 15].

5-aminosalicylic acid (5-ASA) therapy is commonly used in UC patients [20]. It reduces the signs and symptoms of the disease and brings long-term remission [21]. 5-ASA penetrates the colon mucosa easily and reduces the production of prostaglandins [22, 23]. It also restores the expression of µ-protocadherin (a protein expressed by colorectal epithelial cells that is downregulated upon malignant transformation) and promotes the sequestration of β catenin (a protein involved in the regulation and coordination of cell-cell adhesion and gene transcription) to the plasma membrane [24–27]. Although previous research has suggested that treatment with 5-ASA might be chemo-preventive for colorectal cancer [28, 29], the molecular mechanisms underlying the effect of this drug are not entirely understood.

PSC patients with concomitant UC are often additionally treated with 5-ASA. Our previous study [30] using human intestinal epithelial cell lines indicated that 5-ASA therapy can effectively attenuate the expression of miR-155 involved in tumorigenesis. Therefore, this study aimed to examine potential new strategies for the prevention of H-MSI CRC development in individuals with PSC. Given that we have previously found the overexpression of miR-155 in the ascending colon of patients with PSC [14], and that miR-155 can inhibit the expression of MMR genes and suppress genomic stability in CRC [16], we investigated the possible role of miR-155 modulation by 5-ASA in preventing MSI-H.

## Materials and methods

### Cell culture

The human colon carcinoma cell lines CACO-2 (HTB-37™) and HT-29 (HTB-38™) were purchased from the American Type Culture Collection. NCM460D (normal mucosal epithelial cells) were obtained from INCELL Innovative Life Science Solutions (Cell License Material Transfer Agreement #204). All cells were cultured in accordance with the manufacturer’s recommendations and incubated in a humidified atmosphere of 5% CO2 at 37 °C.

### Cell transfection

Transient transfections with miR-155Mimic (Ambion mirVana^®^ miR-155Mimic, hsa-miR-155; ID: MC28440; Thermo Fisher Scientific, Waltham, MA, USA) were performed using Lipofectamine RNAiMAX reagent (Invitrogen, Carlsbad, CA, USA). A standard or reverse transfection protocol was selected based on preliminary experiments according to cell type, high transfection efficiency and low cellular toxicity. Cells with Lipofectamine (vehicle-treated cells) were used as the control group for transfected cells. In a standard transfection protocol, the cells were seeded into a 6-well plate and transfected on day two. In the reverse transfection protocol, cells were added directly to a 6-well plate containing a mixture of transfection solutions of miR-155Mimic, Lipofectamine RNAiMAX and Opti-MEM Reduced Serum Medium (Gibco, Paisley, UK).

### Cell treatments

To investigate the effect of 5-ASA (5-ASA, mesalamine 99%, ID: A3537-25G; Sigma-Aldrich, Saint Louis, MO, USA) on MMR via miR-155, cells were exposed to 5-ASA (1000 µM dose in CACO-2 and HT-29 cells and 200 µM in NCM460D) for the next 24 h and 48 h after transfection with miR-155Mimic. An appropriate dose of 5-ASA (200–1000 µM) was chosen based on colorimetric MTT assays conducted in every cell line. 5-ASA dissolved as a 100 mM stock solution in DMSO (Sigma-Aldrich, St. Louis, MO, USA; Cat #D2650-5 × 5ML, CAS: 67-68- 5) was protected from light according to the manufacturer’s instructions. Seventy-two hours after transfection, the cells were lysed and stored at − 80 °C until molecular analyses could be conducted. Experiments were repeated at least three times, and the untreated cells were used as negative controls for 5-ASA stimulated/non-transfected cells.

For lipopolysaccharide (LPS) experiments, CACO-2 cells were treated with 1, 5 and 10 µg/ml of LPS. The LPS doses were selected based on literature data [31–34]. After 24 h, the cells were harvested, washed with PBS, and centrifuged for 5 min at 800 rpm. Cell pellets were collected and stored at -80 °C.

### RNA and miRNA expression analysis

Total RNA was extracted from cell pellets using the RNeasy Mini kit (Qiagen, Hilden, Germany) according to the manufacturer’s protocol. For further gene expression analysis, cDNA synthesis was carried out using the SuperscriptTM IV RT kit (Invitrogen, Thermo Fisher Scientific) and miRNA cDNA was synthesised using the TaqMan Advanced miRNA cDNA synthesis kit (Applied Biosystems, Waltham, MA, USA). TaqMan Gene Expression assays were used to measure the transcripts of MLH1 (Hs00179866_m1), MSH2 (Hs00954125_m1), MSH6 (Hs00943000_m1) and the reference 18 S ribosomal RNA (Hs99999901_s1). The expression of miR-155 (002623_mir) and miR-16 (477860_mir) used as endogenous controls were measured using TaqMan Advanced miRNA assays and TaqMan Fast Advanced Master Mix (Applied Biosystems). Data were analysed using 7500 software v2.0.2. (Applied Biosystems) and the relative amounts of transcripts were calculated using the 2^-ΔΔCt^ method.

### Immunoblotting

Proteins were extracted from cell pellets by homogenisation with lysis buffer (RIPA buffer) supplemented with protease inhibitors (Roche, Basel, Switzerland) and phosphatases (PhosSTOP EASYpack; Roche, Basel, Switzerland). A total of 30 µg of proteins were used in the experiments. Proteins were electrophoresed on 10% SDS-polyacrylamide gels and applied to a PVDF polyvinylidene membrane (Thermo Scientific, Rockford, IL, USA) under semi-dry transfer conditions (Hoefer, Inc., Holliston, MA, USA). After blocking with 5% skimmed milk, the membranes were incubated for 2.5 h at room temperature with primary antibodies MLH1: 4C9C7 (Cell Signaling Technology, Inc., Danvers, MA, USA), MSH2: D24B5 (Cell Signaling Technology, Inc.), and MSH6: 3E1 (Cell Signaling Technology, Inc.) at a concentration of 1:2000. They were then incubated with peroxidase-conjugated anti-mouse secondary antibodies (1:5000) (Jackson ImmunoResearch Laboratories, Inc, code: 115-035-146) or anti-rabbit secondary antibodies (1:5000) (Boster antibody and ELISA experts, code: BA1054). Protein loading was normalised to anti-glyceraldehyde 3-phosphate dehydrogenase (GAPDH) (1:5000, sc-25,778 + HRP; Santa Cruz). Bands were visualised using a chemiluminescence detection system (Chemiluminescent HRP Substrate, Millipore, MA, USA) and quantified using the MicroChemi 2.0 system and GelQuant software (Maale HaHamisha, Jerusalem, Israel).

### Statistical analysis

StatView software version 5.0 (SAS Institute, Cary, NC, USA) and GraphPad Prism version 7.0 software (GraphPad Software, San Diego, CA, USA) were used for the statistical analyses. Comparisons between groups were performed with one-way analysis of variance (ANOVA) or the non-parametric Mann-Whitney test. All graphs were generated using GraphPad Prism. Data are represented as mean ± standard error of the mean from at least three independent experiments. A p-value < 0.05 was considered statistically significant (*, *p* < 0.05; **, *p* < 0.01; ***, *p* < 0.005; and ****, *p* < 0.001).

## Results

### LPS-induced miR-155

**Fig. 1:**
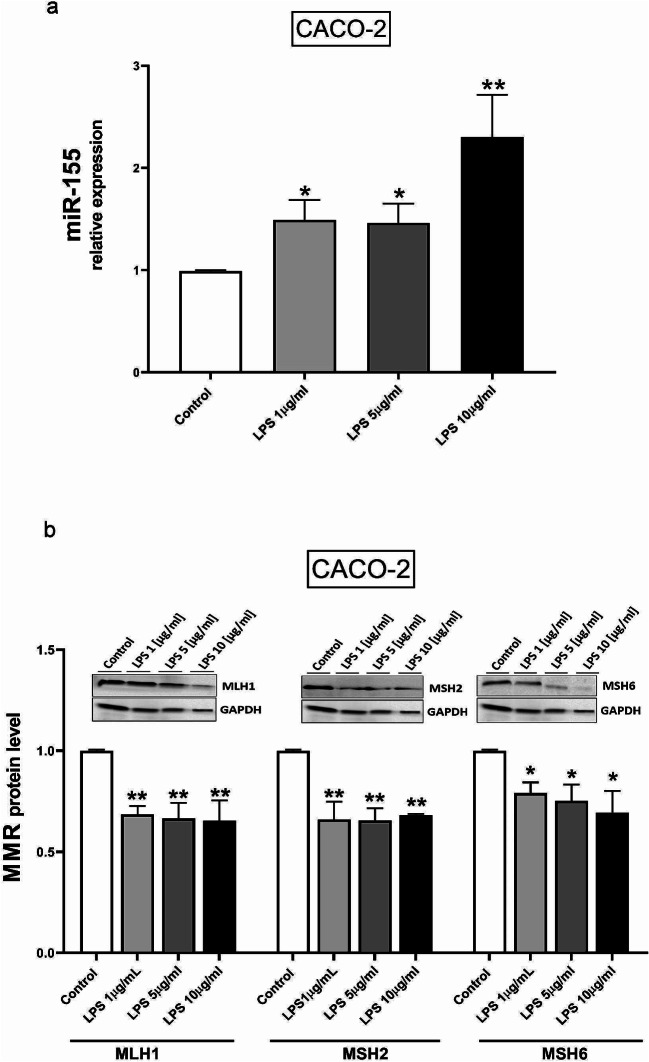
The elevated expression of miR-155 and the reduced level of MSH2, MLH1 and MSH6 proteins in CACO-2 cells after LPS treatment. The expressions of miR-155 in CACO-2 cells after treatment with different doses of LPS (1, 5 and 10 µg/ml) were measured by quantitative PCR (real-time PCR) (**a**). Western blot analysis revealed MSH2, MLH1 and MSH6 protein levels in CACO-2 cells treated with LPS (1, 5 and 10 µg/ml) (**b**). Results are presented as mean ± standard error of the mean (*n* = 3); *p < 0.05, ** < 0.01 vs. controls

Since miR-155 is upregulated in PSC [14] and inflammation is associated with PSC development, we first tested whether LPS as a pro-inflammatory agent could affect the expression of miR-155 in human intestinal CACO-2 cells. For this purpose, cells were incubated for 24 h with different doses of LPS, i.e., 1, 5 and 10 µg/ml. The analysis showed that the expression of miR-155 led to a 1.4-fold increase with both 1 and 5 µg/ml of LPS (1.49 ± 0.19, *p* = 0.042 and 1.46 ± 0.189, *p* = 0.046, respectively) and a 2.3-fold increase with 10 µg/ml of LPS (2.3 ± 0.411, *p* = 0.005) (Fig. 1a). As the close link between miR-155 and the MMR system has been previously demonstrated [16], we checked how miR-155 influenced the levels of MMR genes in LPS-stimulated CACO-2 cells. After 24 h of CACO-2 incubation with 1, 5 and 10 µg/ml of LPS, we investigated the expression of MMR genes, including MLH1, MSH2, and MSH6 at the mRNA (Fig. S1), and protein levels. An immunoblot analysis showed that all doses of LPS, i.e., 1, 5 and 10 µg/ml, significantly diminished the level of MLH1 (0.6 ± 0.04, *p* = 0.01; 0.6 ± 0.04, *p* = 0.007; and 0.6 ± 0.1, *p* = 0.006, respectively), MSH2 (0.6 ± 0.08, *p* = 0.002; 0.6 ± 0.06, *p* = 0.001; and 0.6 ± 0.07, *p* = 0.002, respectively) and MSH6 proteins (0.79 ± 0.05, *p* = 0.04; 0.75 ± 0.08, *p* = 0.04; and 0.69 ± 0.1, *p* = 0.017, respectively) (Fig. 1b).

### miR-155 significantly downregulated core MMR proteins

**Fig. 2:**
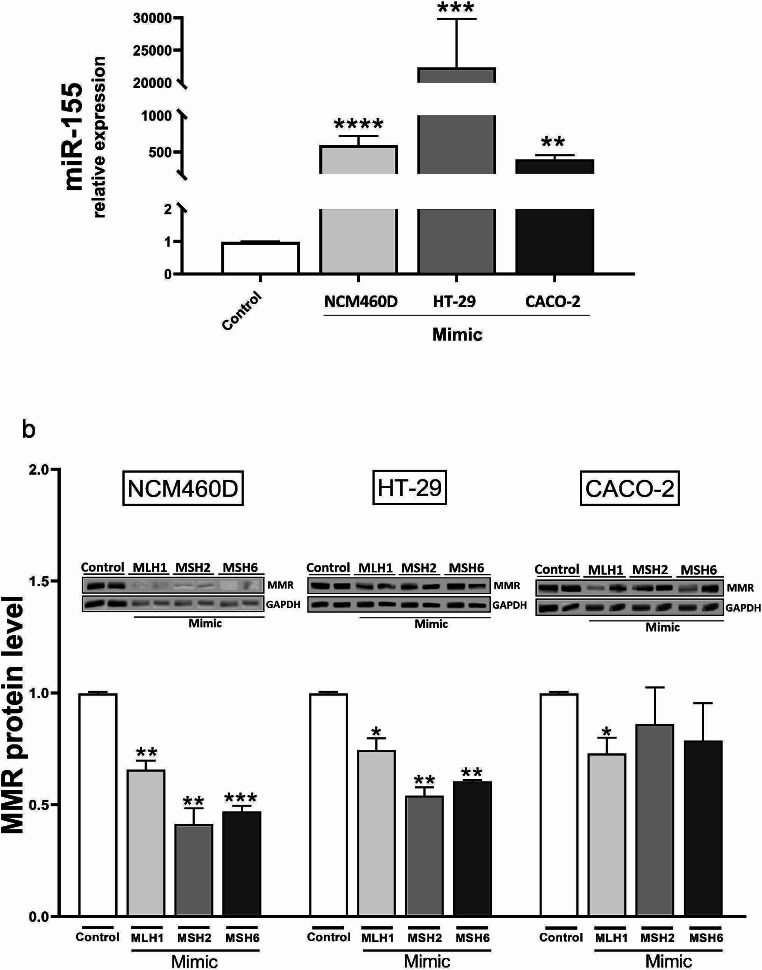
The effect of miR-155 overexpression on MMR protein levels. The upregulation of miR-155 after transient transfection with miR-155-5pMimic molecules was confirmed in different colon cell lines: NCM460D, HT-29 and CACO-2 (**a**). NCM460D and HT-29 cells responded to miR-155 overexpression and decreased MLH1, MSH2 and MSH6 protein levels were observed. In CACO-2 cells, only MLH1 responded to miR-155-5pMimic (**b**). Data are present as mean ± standard error of the mean. Gene expression levels of micro RNA were normalised to the reference miR-16, and the levels of each protein were normalised to GAPDH. Statistical analyses were performed using ANOVA or the Mann-Whitney test. Mimic (miR-155-5pMimic).*p < 0.05 vs. controls, **p < 0.01 vs. controls, ***p < 0.001 vs. controls, ****p < 0.0001 vs. controls

To determine the relationship between miR-155 and the DNA MMR system, transient transfection with miR-155Mimic was performed, and the levels of MLH1, MSH2 and MSH6 mRNA were examined. Successful transfection was confirmed in normal colon (NCM460D (*p* < 0.0001)) and tumorigenic cells (HT-29, *p* = 0.0002; and CACO-2 (*p* = 0.001) (Fig. 2a). Previous results [14] have shown that, in NCM460D, the response to miR-155 overexpression leads to significant inhibition of MLH1, MSH2,and MSH6 mRNA levels compared to non-transfected cells. In this study, we conducted further experiments and examined the MMR protein levels after transfection of miR-155Mimic (Fig. 2b). In non-tumorigenic NCM460D cells, the response to miR-155 led to a significant downregulation of MLH1 (1.03 ± 0.04 in control (CRT) vs. 0.7 ± 0.04 in miR-155Mimic, *p* = 0.002), MSH2 (1.03 ± 0.04 in CRT vs. 0.4 ± 0.07 in miR-155Mimic, *p* = 0.002) and the MSH6 protein (1.03 ± 0.04 in CRT vs. 0.5 ± 0.03 in miR-155Mimic, *p* = 0.0002) (Fig. 2b). The downregulation of the MMR protein was also observed in tumorigenic HT-29 cells (1.0 ± 0.06 in CRT vs. 0.7 ± 0.05 in miR-155Mimic, *p* = 0.03 for MLH1; 1.0 ± 0.06 in CRT vs. 0.5 ± 0.04 in miR-155Mimic, *p* = 0.003 for MSH2; and 1.0 ± 0.06 in CRT vs. 0.6 ± 0.01 in miR-155Mimic, *p* = 0.002 for MSH6). In contrast, in CACO-2 cells, the enhanced expression of miR-155 was accompanied by a downregulation of only one of the MMR proteins, i.e., MLH1 (1.0 ± 0.06 in CRT vs. 0.7 ± 0.07 in miR-155Mimic, *p* = 0.04)(Fig. 2b).

### 5-ASA effectively induces MMR via miR-155

**Fig. 3:**
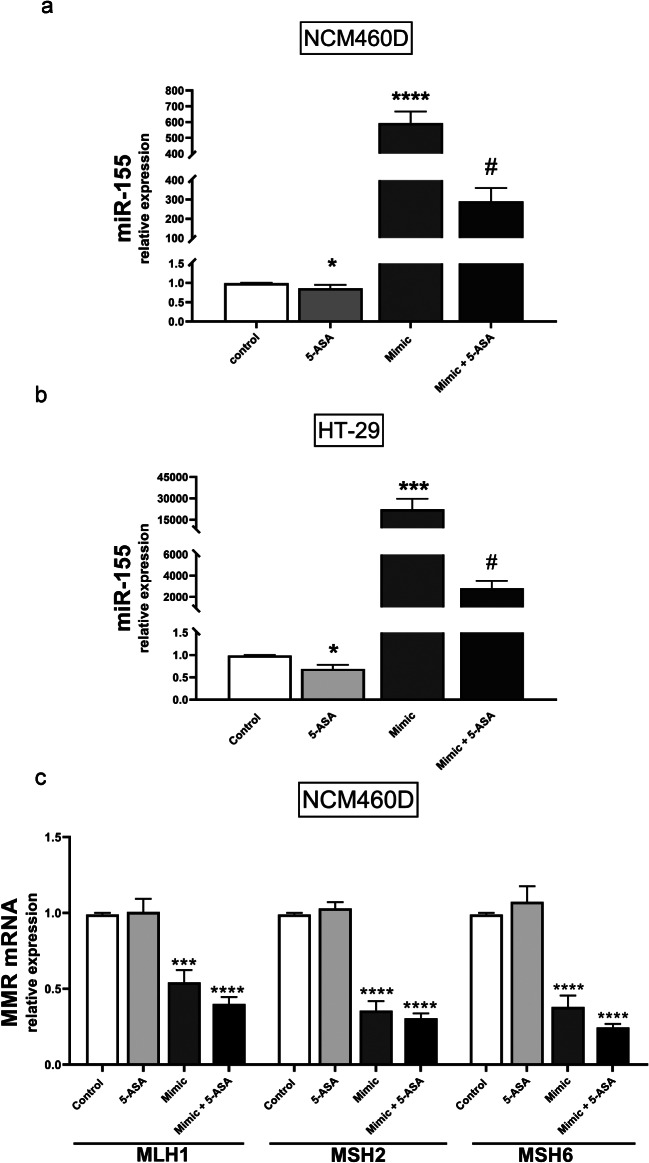
The effect of 5-ASA on miR-155 and MMR gene expression MicroRNA-155 expression decreased significantly after exposure to 5-ASA in transfected and non-transfected NCM460D (**a**) and HT-29 cells (**b**). The MMR genes’ (MLH1, MSH2 and MSH6) relative expression was reduced after 5-ASA with or without miR-155-5pMimic in NCM460D cells (**c**). Data are present as mean ± standard error of the mean. Micro RNA expression levels were normalised to the reference miR-16. Statistical analyses were performed using ANOVA or the Mann-Whitney test. Mimic (miR-155-5pMimic). *p < 0.05 vs. controls, **p < 0.01 vs. controls, ***p < 0.001 vs. controls, ****P < 0.0001 vs. controls, # *p* < 0.05 vs. Mimic

Recently, we showed that the expression level of miR-155 following 24 h treatment with 5-ASA was significantly suppressed in CACO-2 cell lines [30]. This phenomenon was confirmed in NCM460D and HT-29 cell lines in this study. We found that in normal colon cells, 5-ASA significantly reduced both the basal level of miR-155 expression (1.03 ± 0.03 in CRT vs. 0.9 ± 0.1 in 5-ASA, *p* = 0.02; Fig. 3a) and after transfection with miR-155Mimic (593.7 ± 73.6 in miR-155Mimic vs. 289.8 ± 69.2 in 5-ASA with miR-155Mimic, *p* = 0.001; Fig. 3a). In HT-29 cells, the expression of miR-155 was also reduced after 5-ASA exposure in miR-155 transfected cells (22350.2 ± 7470.9 in miR-155Mimic vs. 2818.6 ± 690.4 in 5-ASA with miR-155Mimic, *p* = 0.0006) and non-stimulated cells (1.03 ± 0.03 in CRT vs. 0.7 ± 0.1 in 5-ASA, *p* = 0.03) (Fig. 3b). These results prompted us to further investigate the functional role of 5-ASA in MMR modulation.

In normal colon cells, miR-155 overexpression led to a significant downregulation of MLH1 (1.03 ± 0.03 in CRT vs. 0.5 ± 0.05 in miR-155Mimic, *p* = 0.0002), MSH2 (1.03 ± 0.03 in CRT vs. 0.4 ± 0.06 in miR-155Mimic, *p* = 0.0001) and MSH6 mRNA levels (1.03 ± 0.03 in CRT vs. 0.4 ± 0.1 in miR-155Mimic, *p* < 0.0001) (Fig. 3c). This reduction was also evident following 5-ASA treatment in cells transfected with miR-155Mimic (MLH1: 1.03 ± 0.03 in CRT vs. 0.4 ± 0.05 in 5-ASA with mimic 155, *p* < 0.0001; MSH2: 1.03 ± 0.03 in CRT vs. 0.3 ± 0.03 in 5-ASA with miR-155Mimic, *p* < 0.0001; and MSH6 mRNA levels: 1.03 ± 0.03 in CRT vs. 0.2 ± 0.02 in 5-ASA with miR-155Mimic, *p* < 0.0001).

An analysis of the relative expression of MMR genes in the HT-29 and CACO-2 cells (Fig. S2) exhibited no differences; therefore, we conducted further experiments at the MMR protein level in NCM460D, HT-29 and CACO-2 cell lines.

**Fig. 4:**
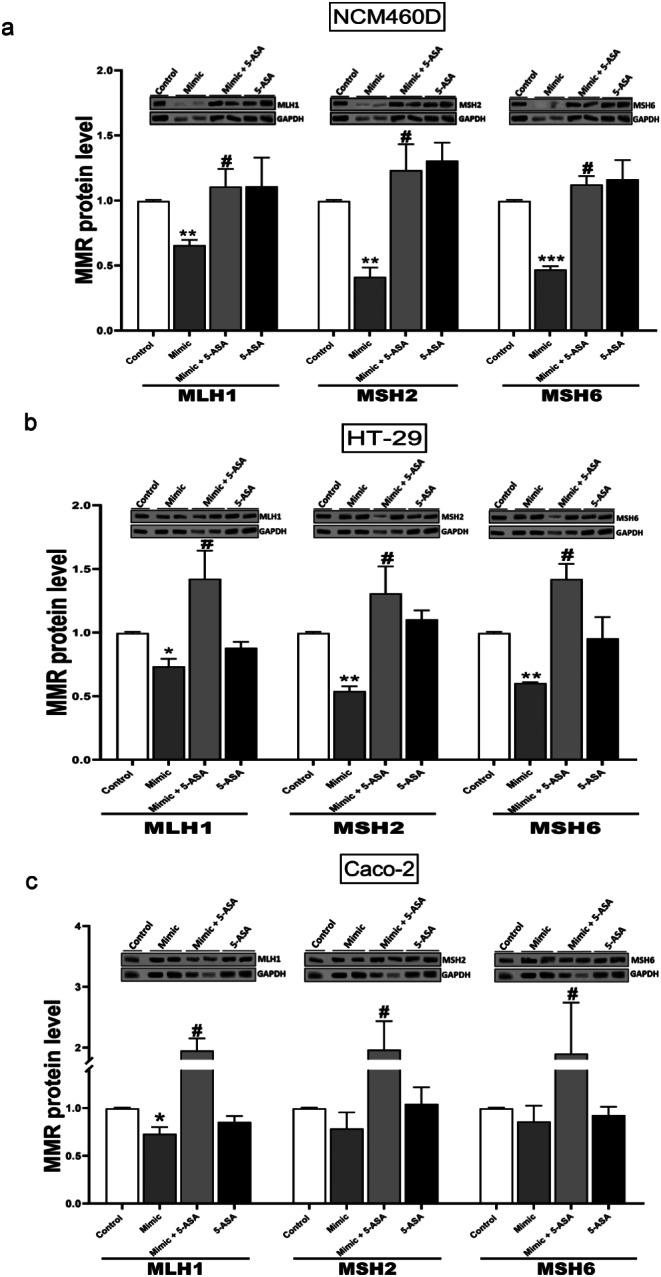
Levels of MLH1, MSH2 and MSH6 proteins after 5-ASA with or without miR-155Mimic transfection in intestinal epithelial cells. 5-ASA restored MMR protein level after induction of miR-155 in NCM460D (**a**) HT-29 (**b**) and CACO-2 (**c)**. Data are presented as mean ± standard error of the mean. The levels of each protein were normalised to GAPDH. Statistical analyses were performed using ANOVA or the Mann-Whitney-test. Mimic (miR-155-5pMimic). *p < 0.05 vs. controls, **p < 0.01 vs. controls, ***p < 0.001 vs. controls, ****p < 0.0001 vs. controls, # p < 0.05 vs. Mimic

The immunoblot analysis confirmed the influence of 5-ASA on the MSI profile. 5-ASA blocked the miR-155-induced inhibition of MLH1 (1.12 ± 0.13 in 5-ASA with miR-155Mimic vs. 0.7 ± 0.04 in miR-155Mimic, *p* = 0.007), MSH2 (1.12 ± 0.2 in 5-ASA with miR-155Mimic vs. 0.4 ± 0.07 in miR-155Mimic, *p* = 0.002) and MSH6 (1.12 ± 0.07 in 5-ASA with miR-155Mimic vs. 0.5 ± 0.03 in miR-155Mimic, *p* = 0.001) in NCM460D cells (Fig. 4a). Similarly to normal colon cells, the overexpression of miR-155 in the HT-29 cell line (Fig. 4b) led to the strong downregulation of MMR, which was reversed by 5-ASA treatment. Thus, all examined MMR protein levels, MLH1 (0.7 ± 0.07 in miR-155Mimic vs. 1.4 ± 0.22 in 5-ASA with miR-155Mimic, *p* = 0.004), MSH2 (0.05 ± 0.04 in miR-155Mimic vs. 1.3 ± 0.2 in 5-ASA with miR-155Mimic, *p* = 0.002) and MSH6 (0.6 ± 0.006 in miR-155Mimic vs. 1.4 ± 0.1 in 5-ASA with miR-155Mimic, *p* = 0.0014) was increased in miR-155Mimic positive cells after 5-ASA co-treatment. Results from the CACO-2 cell line (Fig. 4c) showed that 5-ASA treatment elevated levels of MLH1 (0.7 ± 0.07 in miR-155Mimic vs. 1.9 ± 0.8 in 5-ASA with miR-155Mimic, *p* = 0.05), MSH2 (0.86 ± 0.17 in miR-155Mimic vs. 2.0 ± 0.2 in 5-ASA with miR-155Mimic, *p* = 0.005) and MSH6 (0.8 ± 0.2 in miR-155Mimic vs. 2.0 ± 0.5 in 5-ASA with miR-155Mimic, *p* = 0.03) in miR-155 transfected cells. 5-ASA alone did not change the protein levels of MLH1, MSH2 or MSH6 in any of the colon cell lines. Thus, 5-ASA’s effect on MMR is manifested through the mechanism of the miR-155 pathway. Our results uncovered no significant differences in MMR mRNA levels in the colon cell lines (Fig. S3).

## Discussion

Our in vitro study using human intestinal epithelial cells has indicated that 5-ASA can potentially inhibit miR-155 expression induced by inflammation imitated by LPS treatment or by transient transfection with miR-155Mimic. Moreover, we have shown that co-treatment with 5-ASA not only suppressed miR-155 but also led to the increased expression of MMR protein levels. This study highlights the chemo-protective effects of 5-ASA, which can potentially be used in the colon of patients with PSC.

Patients with PSC have an increased risk of developing primary bile duct cancer and CRC [5, 35]. The risk of developing CRC in PSC patients with concurrent UC is 14% at 10 years and 31% at 20 years, compared to a steady risk of 2.3% in patients without concurrent UC [35, 36]. Prolonged exposure to high levels of bile acids can lead to the generation of genomic instability, the development of apoptosis resistance and, ultimately, cancer [37]. Our previous study confirmed the effect of toxic lithocholic and glycochenodeoxycholic acids or LPS on miR-155 expression in HT-29 and NCM460D cell lines [14] and human cholangiocytes [38]. Furthermore, it has been postulated that the expression of miR-155 is increased in inflammation-induced cancer cells [39]. In this study, we found that miR-155 expression was significantly elevated in human intestinal epithelial CACO-2 cells incubated with LPS. Our study demonstrates that in LPS-treated CACO-2 cells, miR-155 is overexpressed and associated with reduced MMR protein levels. The overexpression of miR-155 after LPS treatment was previously demonstrated in mouse and human cells [39], which suggests that this microRNA plays a role in the innate immune response.

In our previous study, we transiently transfected human epithelial cell lines with miR-155Mimic to investigate the relationship between MMR mRNA expression and miR-155 [14]. The results from normal colonic cells (NCM460D) confirmed that, similar to normal, non-tumorigenic tissue, the upregulation of miR-155 results in the significant downregulation of MLH1, MSH2 and MSH6 mRNA expression [14]. However, in human colon adenocarcinoma cells (HT-29), the response to miR-155 overexpression is less pronounced [14]. It has been speculated that the negative regulation of MMR expression by miRNA is due to translational inhibition [16]. It is not unusual that an abundance of mRNA transcripts does not reliably predict changes in protein expression. Thus, an analysis of protein levels is important for providing a functional context to interpret genomic abnormalities [40]. Therefore, in this study, we analysed the effect of miR-155 overexpression on the protein levels of MLH1, MSH2 and MSH6 in three human intestinal epithelial cells (NCM460D, CACO-2 and HT-29). We confirmed that miR-155 may regulate components of the MMR machinery [14] and, consequently, rates of MSI [41–43].

We further examined a possible mechanism of 5-ASA action. Recent studies have proposed 5-ASA as a candidate compound for chemo-prevention due to a reduction in the incidence and multiplicity of intestinal tumours in Msh2 ^loxP/loxP^ Villin-Cre mice [44]. To gain insight into the molecular effects of 5-ASA during tumorigenesis, the expressions of miR-155, MMR mRNA and proteins were evaluated after drug exposure in miR-155-transfected human epithelial cell lines. The miR-155-dependent effect of 5-ASA on MLH1, MSH2 and MSH6 proteins was confirmed in three cell lines. These novel observations have not been previously reported. Other authors have observed the effect of 5-ASA on miR-206 expression [45]. An analysis of colon biopsy tissues has uncovered significantly lower expression of miR-206 in UC patients who received a higher dose of 5-ASA. Those results were confirmed in the human colon cancer cell line-HT-29, as an almost two-fold decrease in miR-206 expression was observed 4 h after 5-ASA treatment.

Interestingly, our study clearly shows that, in contrast to CACO-2 and HT-29 cell lines, in NCM460D cells, both mRNA and protein levels of MMR were modulated by 5-ASA. Apart from the various dominant cancer cells used for in vitro studies, normal human cell lines are of particular importance in the context of CRC [46]. The value of the non-tumorigenic NCM460 cell model as a control in anti-tumour strategies targeting colon adenocarcinoma has been previously described [47]. Our findings have established the suitability of NCM460D cells as an in vitro model system for investigating the details of drug-miRNA-MMR pathways.

MSI-H CRCs are associated with many diseases, including Lynch syndrome (caused by autosomal dominant mutations to the major MMR genes MLH1, MSH2, MSH6 or PMS2 and the EPCAM gene that inactivates MSH2) [49, 50]. We previously demonstrated the involvement of miR-155 in chronic inflammation in the colons of PSC-UC patients and the relationship with MSI-H CRC markers [14]. In this study, for the first time, we show the drug-miRNA-MMR relationship in colon cell lines in vitro. A phase II clinical trial using 2000 mg mesalamine (5-ASA) for the prevention of colorectal neoplasia in Lynch Syndrome patients is currently underway [51]. If this trial confirms the effectiveness of 5-ASA against colorectal neoplasia in genetically-induced Lynch Syndrome, then (taking into consideration our results from this in vitro study) we would suggest a new strategy of testing all PSC patients for an endpoint of MSI-H, and patients with a known MSI status should receive 5-ASA as a preventive therapy.

In conclusion, this research focus on the effects of 5-ASA treatment on the MMR system. Our in vitro study indicates that 5-ASA therapy can effectively attenuate the expression of miR-155 and restore MMR protein expression inhibited by miR-155.

## Electronic supplementary material

Below is the link to the electronic supplementary material.

Supplementary Material 1

## Data Availability

No datasets were generated or analysed during the current study.
