# Supplementary material for: Modulation of miR-155-5p signalling via 5-ASA for the prevention of high microsatellite instability: an in vitro study using human epithelial cell lines
Source: J Physiol Biochem. 2024 Jul 10;80(3):573–83. doi: 10.1007/s13105-024-01033-y (PMC11502576; doi:10.1007/s13105-024-01033-y)
Supplement: Supplementary file 1 — Supplementary Material 1 [file 13105_2024_1033_MOESM1_ESM.pdf]

LPS at the dose of 5µg/ml significantly elevated the expression of both MLH1 and MSH2 genes (2.5 ± 0.9, p=0.04 and 2.3 ± 0.7, p=0.04; respectively) while reduced MSH6 gene expression (0.51 ± 0.21, p=0.04) in CACO-2 cells.

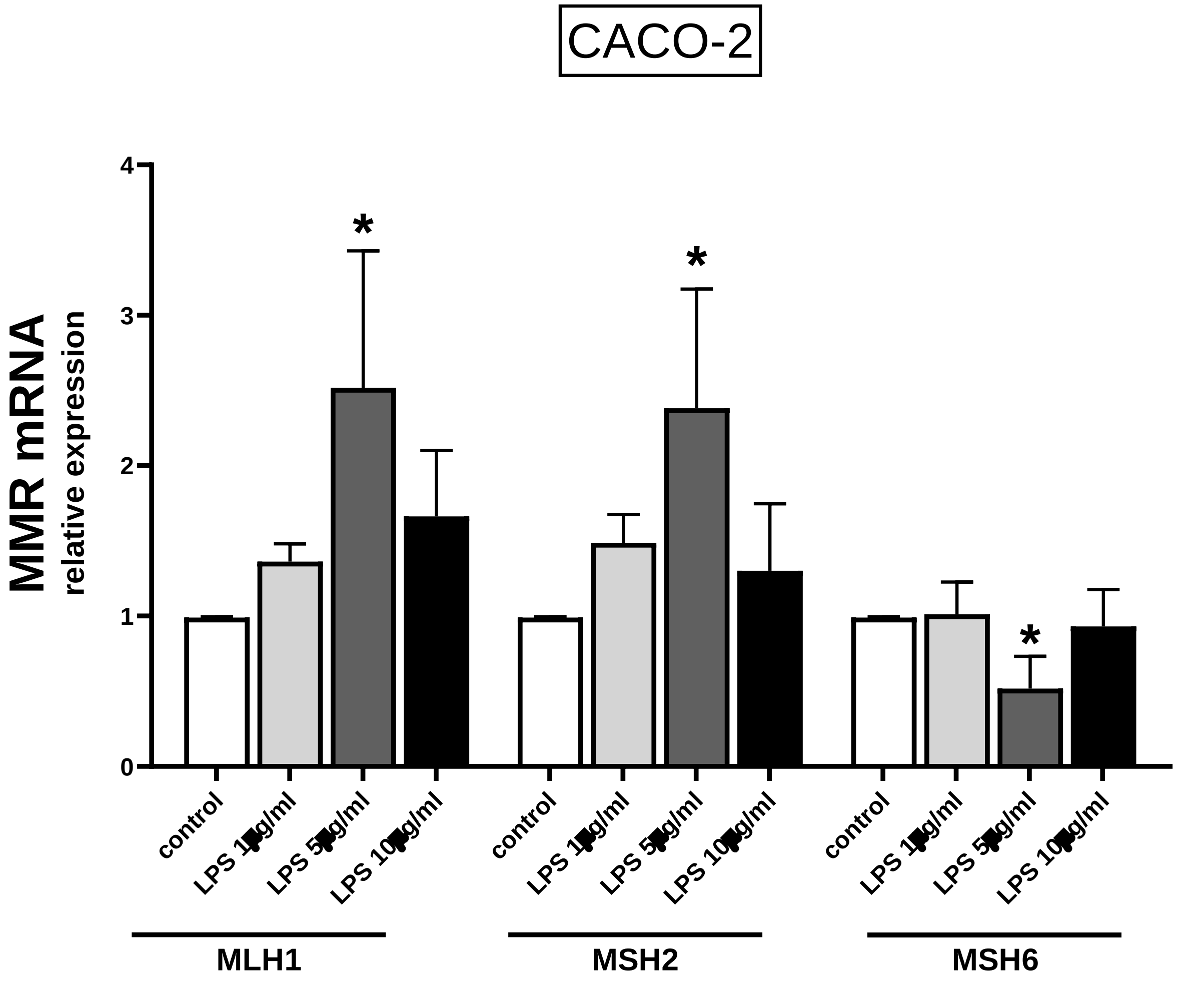

Figure S1. The expressions of MLH1, MSH2, and MSH6 in CACO-2 cells after treatment with different doses of LPS (1, 5, and 10µg/ml) were measured by quantitative PCR (real-time PCR). Levels of gene expression were normalised to the reference miR-16. Statistical analysis was performed using ANOVA or Mann-Whitney-test. Results are presented as mean ± SEM (n=3); **p<0.05 vs. controls.*

The analysis of relative expression of MMR genes in tumorigenic cells CACO-2 showed no differences.

a

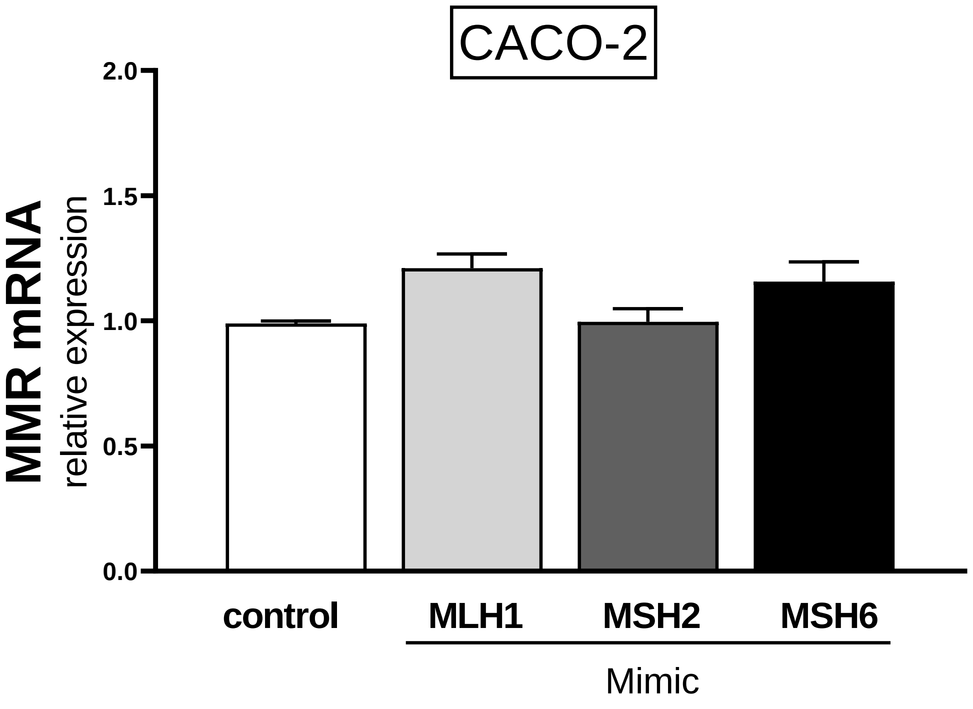

Figure S2. Induction of miR-155 by miR-155Mimic did not affect MMR genes expression mRNA level in CACO-2. Data are present as mean ± SEM. Levels of gene expression were normalised to the reference miR-16. Statistical analysis was performed using ANOVA or Mann-Whitney-test. Mimic (miR-155Mimic).

The relative expression of miR-155 in tumorigenic cells: HT-29 and CACO-2. The results uncovered no significant difference in MMR mRNA level. Notably, 5-ASA, which initially reduced the expression of miR-155 in the NCM460D and HT-29 cell, did not affect MMR genes at mRNA level.

a

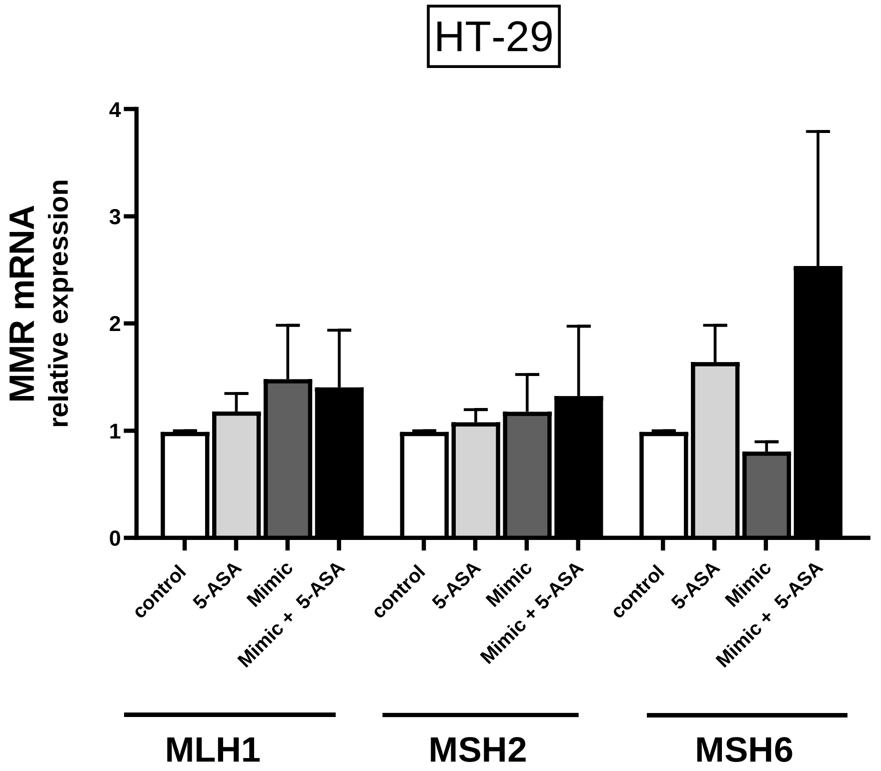

b

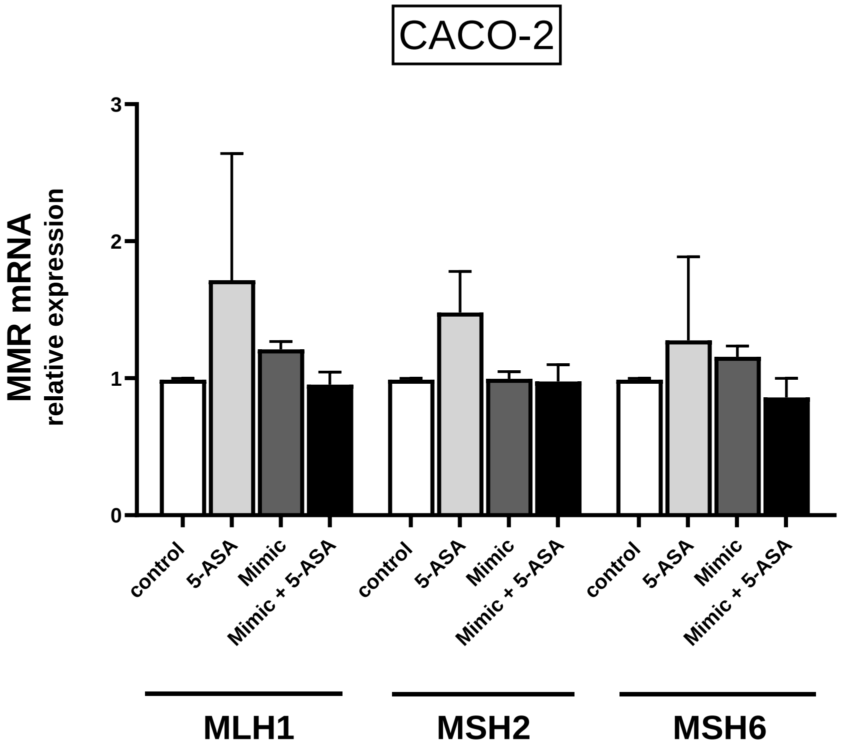

Figure S3. The MMR genes (MLH1, MSH2, MSH6) relative expression after 5-ASA with or without miR-155Mimic in HT-29 (**a**) or CACO-2 (**b**). Levels of gene expression were normalised to the reference miR-16. Statistical analysis was performed using ANOVA or Mann-Whitney-test. Results are presented as mean ± SEM. Mimic (miR-155Mimic).
